# Murine K_2P_5.1 Deficiency Has No Impact on Autoimmune Neuroinflammation due to Compensatory K_2P_3.1- and K_V_1.3-Dependent Mechanisms

**DOI:** 10.3390/ijms160816880

**Published:** 2015-07-24

**Authors:** Stefan Bittner, Nicole Bobak, Majella-Sophie Hofmann, Michael K. Schuhmann, Tobias Ruck, Kerstin Göbel, Wolfgang Brück, Heinz Wiendl, Sven G. Meuth

**Affiliations:** 1 Department of Neurology, University of Münster, Albert-Schweitzer-Campus 1, Münster 48149, Germany; E-Mails: majella.hofmann@uni-muenster.de (M.-S.H.); tobias.ruck@ukmuenster.de (T.R.); kerstin.goebel@ukmuenster.de (K.G.); heinz.wiendl@ukmuenster.de (H.W.); sven.meuth@ukmuenster.de (S.G.M.); 2 LabEx ICST, Institut de Pharmacologie Moléculaire et Cellulaire, CNRS and Université de Nice-Sophia Antipolis, Valbonne 06560, France; E-Mail: nicole.bobak@txcell.com; 3 Department of Neurology, University of Würzburg, Würzburg 97070, Germany; E-Mail: Schuhmann_M@ukw.de; 4 Department of Neuropathology, University Medical Center, Georg August University, Göttingen 37073, Germany; E-Mail: neuropat@med.uni-goettingen.de; 5 Department of Physiology I-Neuropathophysiology, University of Münster, Münster 48149, Germany

**Keywords:** ion channels, potassium channels, K2P channels, K_2P_5.1, TASK2, KCNK5, autoimmune neuroinflammation, multiple sclerosis, EAE

## Abstract

Lymphocytes express potassium channels that regulate physiological cell functions, such as activation, proliferation and migration. Expression levels of K_2P_5.1 (TASK2; KCNK5) channels belonging to the family of two-pore domain potassium channels have previously been correlated to the activity of autoreactive T lymphocytes in patients with multiple sclerosis and rheumatoid arthritis. In humans, K_2P_5.1 channels are upregulated upon T cell stimulation and influence T cell effector functions. However, a further clinical translation of targeting K_2P_5.1 is currently hampered by a lack of highly selective inhibitors, making it necessary to evaluate the impact of KCNK5 in established preclinical animal disease models. We here demonstrate that *K_2P_5.1* knockout (*K_2P_5.1^−^*^/*−*^) mice display no significant alterations concerning T cell cytokine production, proliferation rates, surface marker molecules or signaling pathways. In an experimental model of autoimmune neuroinflammation, *K_2P_5.1^−^*^/*−*^ mice show a comparable disease course to wild-type animals and no major changes in the peripheral immune system or CNS compartment. A compensatory upregulation of the potassium channels K_2P_3.1 and K_V_1.3 seems to counterbalance the deletion of *K_2P_5.1*. As an alternative model mimicking autoimmune neuroinflammation, experimental autoimmune encephalomyelitis in the common marmoset has been proposed, especially for testing the efficacy of new potential drugs. Initial experiments show that K_2P_5.1 is functionally expressed on marmoset T lymphocytes, opening up the possibility for assessing future K_2P_5.1-targeting drugs.

## 1. Introduction

Ion channels have long been established as important regulators of the physiological functions of T lymphocytes and other immune cells [1]. Potassium channels are required to maintain a hyperpolarized membrane potential necessary for sustaining the electrochemical driving force for Ca^2+^ ion entry upon T cell receptor stimulation [2,3]. Targeting of potassium channels as a potential therapeutic strategy for autoinflammatory disorders can nowadays be viewed as an established concept, and the first attempts for clinical trials are ongoing [3,4,5]. While the most intensively-studied potassium channels on lymphocytes are the voltage-gated K_V_1.3 and the Ca^2+^-activated K_Ca_3.1, we and others recently added members of the family of two-pore domain (K_2P_) potassium channels to the picture [4]. K_2P_ channels are mainly voltage-independent K^+^ channels that are modulated by changes in pH, lipid metabolites or hypoxia [6]. Human and murine T lymphocytes express the K_2P_ channels K_2P_3.1 (TASK1; KCNK3), K_2P_5.1 (TASK2; KCNK5) and K_2P_9.1 (TASK3; KCNK9) [1,4,7]. *K_2P_3.1^−^*^/*−*^ mice are less susceptible to the induction of experimental autoimmune encephalomyelitis (EAE), an animal model of multiple sclerosis (MS), and specific pharmacological blockers have a beneficial effect on clinical disease symptoms [8,9]. Mice with a genetic deletion for *K_2P_9.1*, in direct comparison, showed a less pronounced protection against neuroinflammatory damage [8]. In humans, all three K_2P_ channels are involved in T cell functions, such as proliferation or cytokine production [7,10]. Especially expression of K_2P_5.1 is increased following T cell activation and in pathogenic T lymphocytes from patients with MS [10] and rheumatoid arthritis (RA) [11]. An especially pronounced upregulation of K_2P_5.1 was found on T lymphocytes located in the cerebrospinal fluid of MS patients and in the synovial fluid of RA patients, respectively [10,11]. However, the further clinical translation of targeting K_2P_5.1 is at present not achievable due to a lack of highly selective K_2P_5.1-channel inhibitors demonstrating the need for mechanistical studies in established preclinical disease models. The impact of K_2P_5.1 on T lymphocyte function has been predominantly addressed in human cells, except basic physiological studies showing an involvement of K_2P_5.1 in human and murine T cell volume regulation [12,13]. An involvement of K_2P_5.1 in murine immune cell function has been shown for B lymphocytes [14,15]. K_2P_5.1 is expressed in WEHI-231 cells, a B lymphocyte cell line derived from murine lymphoma and primary murine B cells. K_2P_5.1 is upregulated upon B cell receptor stimulation and upon hypoxia-induced HIF-1α activation and regulates the influx of Ca^2+^ ions [14,15]. It will be interesting to learn about in-depth immunological studies in the future to assess the functional importance of K_2P_5.1 for murine B cell function.

We here address the impact of K_2P_5.1 on murine T lymphocytes *in vitro* and in the MOG_35–55_ peptide-induced EAE model using *K_2P_5.1^−^*^/*−*^ mice. Furthermore, we perform pilot experiments to evaluate the possibility of performing pharmacological studies inhibiting K_2P_5.1 in the common marmoset, a non-human primate model for autoinflammatory disorders.

## 2. Results

### 2.1. K_2P_5.1^−/−^ and Wild-Type Mice Show a Comparable Disease Course in the EAE Model

**Figure 1:**
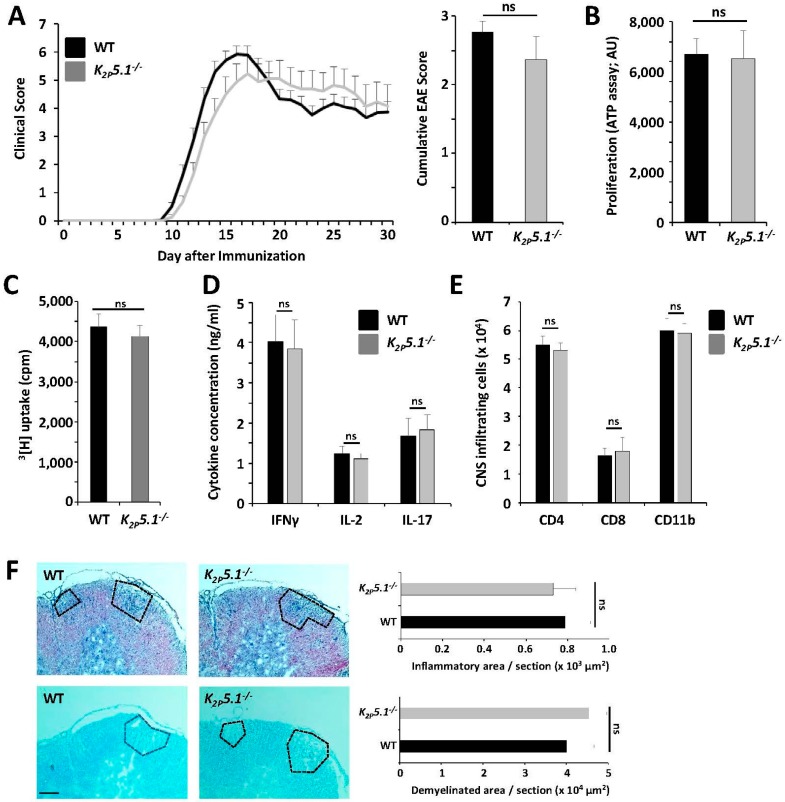
*K_2P_5.1^−^*^/*−*^ and WT mice showed a comparable clinical, immunological and histopathological phenotype in MOG_35–55_ EAE. (**A**) Upon MOG_35-55_ immunization, *K_2P_5.1^−^*^/*−*^ mice showed a comparable clinical disease course (left panel) and cumulative EAE score (right panel) over 30 days compared to wild-type mice (three independent immunizations, each *n* = 7–8); (**B**–**D**) Splenocytes from immunized mice were isolated at disease maximum (d16) and restimulated with 10 µg/mL MOG_35–55_ peptide. No differences were observed for (**B**,**C**) proliferation assessed by two independent methods and for (**D**) the production of IFNγ, IL-2 and IL-17 (*n* = 4); (**E**) Flow-cytometric evaluation of CNS-infiltrating immune cells isolated at disease maximum revealed no significant changes for numbers of CD4^+^, CD8^+^ and CD11b^+^ cells (*n* = 4); (**F**) Histological evaluation of inflammatory infiltrates (HE staining, **upper** panel) and demyelinated area (Luxol fast blue (LFB) staining, **lower** panel) showed no significant differences (*n* = 4–5). Scale bar (100 µm) accounts for all images. ns = not significant.

WT and *K_2P_5.1^−^*^/*−*^ mice were immunized with MOG_35–55_ peptide in order to induce EAE, an animal model mimicking aspects of MS. Both groups showed a comparable disease onset, disease maximum and overall disease course over 30 days (Figure 1A). We performed immunological and histological analysis of EAE mice in order to assess subtle changes not reflected by the clinical disease course. Splenocytes were isolated at disease maximum and restimulated with the same peptide stock used for immunization. No differences were observed for proliferation rates (Figure 1B,C, two independent methods) and for the production of the proinflammatory cytokines IFNγ, IL-2 and IL-17 (Figure 1D). Flow cytometric evaluation of CNS-invading immune cells revealed comparable numbers of CD4^+^ and CD8^+^ T lymphocytes and CD11b^+^ cells (Figure 1E). In agreement, histological evaluation displayed no significant changes for inflammatory infiltrates and demyelinated areas (Figure 1F). In summary, genetic deletion of *K_2P_5.1* resulted in no obvious effect in the EAE model, which is in contrast to the previously-published phenotypes of *K_2P_3.1^−^*^/*−*^ and *K_2P_9.1^−^*^/*−*^ mice [8,9].

### 2.2. K_2P_5.1^−/−^ Mice Show No Obvious Alterations of the Immune System

**Figure 2:**
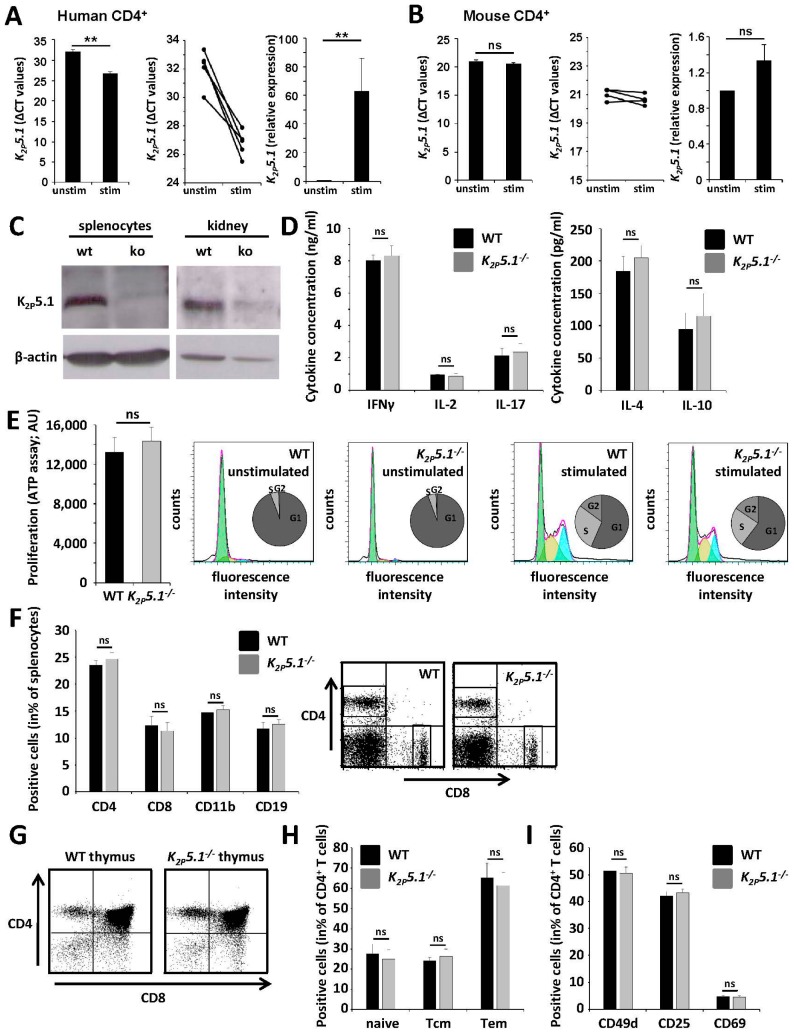
T lymphocytes from *K_2P_5.1^−^*^/*−*^ mice displayed no obvious differences in immune cell function (**A**,**B**). (**A**) Human, but not (**B**) mouse CD4^+^ T lymphocytes show an upregulation of K_2P_5.1 (left panel: mean Δ*C*_t_ values; middle panel: all donors are displayed separately; right panel: relative expression); (**C**) K_2P_5.1 expression can be detected by Western blotting in splenocytes and kidney tissue from wild-type, but not from *K_2P_5.1^−^*^/*−*^ (ko) mice (representative examples); (**D**) WT and *K_2P_5.1^−^*^/*−*^ splenocytes show no differences in cytokine production (*n* = 6); (**E**) No significant differences were observed for proliferation rates from WT and *K_2P_5.1^−^*^/*−*^ T lymphocytes (**left** panel: proliferation assay; **right** panel: flow cytometry-based assessment of cell cycle stages; *n* = 8); (**F**) Splenocytes from WT and *K_2P_5.1^−^*^/*−*^ display a comparable immune cell composition (*n* = 6); (**G**) WT and *K_2P_5.1^−^*^/*−*^ thymi are comparable concerning proportions of double-negative, double-positive and CD4^+^/CD8^+^ single-positive cells (*n* = 4, one representative example is shown); (**H**,**I**) CD4^+^ T lymphocytes from WT and *K_2P_5.1^−^*^/*−*^ mice show no significant alterations concerning (**H**) memory cell composition (**I**) and activation and migration markers (*n* = 5). ns = not significant; **p < 0.05.

It has been reported before that human T lymphocytes upregulate K_2P_5.1 upon T cell receptor (TCR) stimulation [10]. These results were corroborated, as human CD4^+^ T lymphocytes showed an approximately 60-fold upregulation of K_2P_5.1 (Figure 2A). In contrast, while murine lymphocytes also express K_2P_5.1, TCR stimulation only led to a non-significant trend towards an upregulation upon stimulation (Figure 2B). In the next step, we directly compared WT and *K_2P_5.1^−^*^/*−*^ mice. K_2P_5.1 protein was only detected on splenocytes and in kidney tissue of WT, but not of *K_2P_5.1^−^*^/*−*^ animals (Figure 2C). Naive splenocytes were stimulated, yielding no significant differences for cytokine levels of the proinflammatory T_H_1/T_H_17 cytokines IFNγ, IL-2, IL-17, the T_H_2 signature cytokine IL-4 and the regulatory cytokine IL-10 (Figure 2D). In accordance, proliferation rates and cell cycle stages of WT and *K_2P_5.1^−^*^/*−*^ T lymphocytes were comparable (Figure 2E). Furthermore, we addressed a potential influence of *K_2P_5.1* for immune cell development and the composition of splenocytes. Flow cytometric experiments revealed no obvious changes for spleen (Figure 2F) and thymus (Figure 2G). CD4^+^ T lymphocytes from WT and *K_2P_5.1^−^*^/*−*^ mice showed no significant alterations concerning T memory cell composition (Figure 2H) and cell surface markers indicative for cell activation (CD25, CD69) and migratory propensity (CD49d; Figure 2I).

### 2.3. Compensatory Upregulation of K_2P_3.1 and K_V_1.3 in K_2P_5.1^−/−^ T Lymphocytes

**Figure 3:**
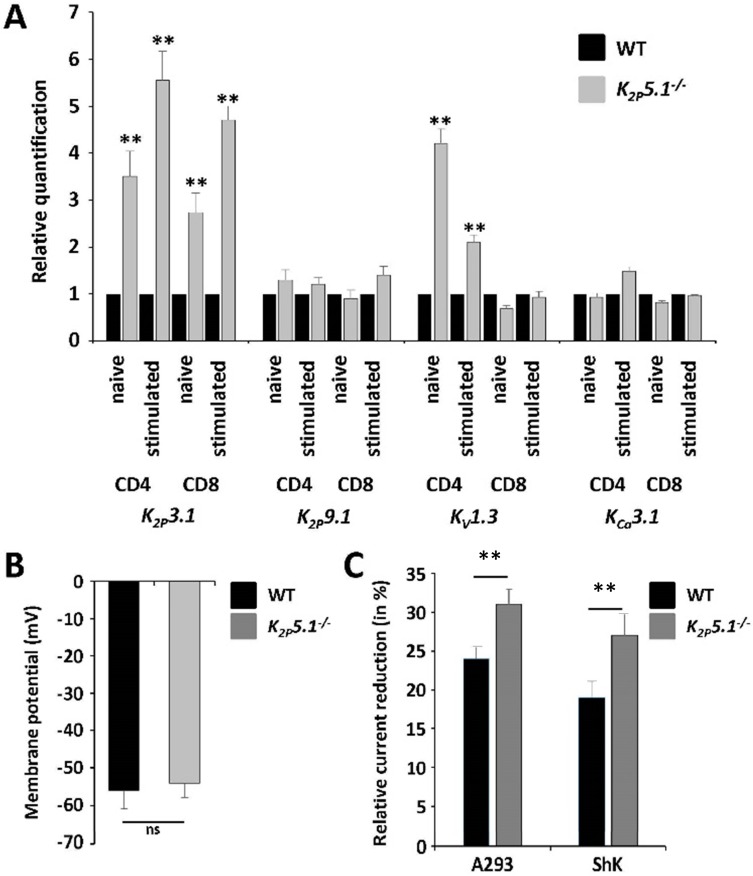

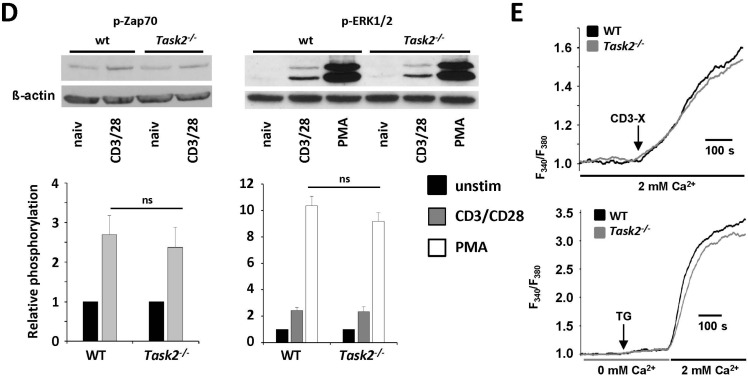
*K_2P_5.1* deletion results in a compensatory upregulation of K_2P_3.1 and K_V_1.3. (**A**) Relative quantification of unstimulated and stimulated WT and *K_2P_5.1^−^*^/*−*^ CD4^+^ and CD8^+^ T lymphocytes for the expression of *K_2P_3.1*, *K_2P_9.1*, *K_V_1.3* and *K_Ca_3.1* (*n* = 5–6); (**B**) Electrophysiological assessment of the resting membrane potential of unstimulated CD4^+^ T lymphocytes (*n* = 10); (**C**) Voltage steps from −80 to +40 mV for 500 ms were used to record outward currents in stimulated WT and *K_2P_5.1^−^*^/*−*^ CD4^+^ T lymphocytes. Current reduction after application of the K_2P_3.1 inhibitor A293 (10 µM) and the K_V_1.3 inhibitor Shk (10 nM) (*n* = 4) is shown; (**D**) Naive WT and *Task2^−^*^/*−*^ CD4^+^ T lymphocytes were stimulated for 10 min either with CD3/CD28 antibodies or PMA followed by cell lysis and Western blot analysis with antibodies against phosphorylated Zap70 (p-Zap70, left panel) and ERK1/2 (right panel). Representative examples (upper panel) and quantitative evaluation (lower panel, *n* = 4) are shown; (**E**) Calcium imaging experiments using Fura-2 in WT and *Task2^−^*^/*−*^ CD4^+^ T lymphocytes were performed under two conditions: T cell-receptor crosslink (CD3-X) in 2 mM Ca^2+^ (upper panel) or application of thapsigargin (TG) for intracellular Ca^2+^ store depletion in 0 mM Ca^2+^ prior to switching to 2 mM Ca^2+^ solution (lower panel). One out of five representative measurements are shown. ns = not significant; ** *p* < 0.05.

Several potassium channels have been shown to be expressed in murine T lymphocytes [1]. While these channels display unique properties, such as diverse expression patterns, biophysical properties or signaling pathways, they also share basic principles, as they regulate Ca^2+^ influx by setting the membrane potential, important for downstream pathways [16]. Therefore, we searched for a compensatory upregulation of other known potassium channels in *K_2P_5.1^−^*^/*−*^ T lymphocytes. Indeed, K_2P_3.1 was upregulated on both CD4^+^ and CD8^+^ T lymphocytes, and K_V_1.3 was upregulated on CD4^+^ T lymphocytes (Figure 3A). We performed electrophysiological measurements to assess the functional contribution of K_2P_3.1 and K_V_1.3 upon *K_2P_5.1* deletion. No significant differences were observed for the membrane potential, pointing towards a comparable contribution of the whole-cell potassium outward current (Figure 3B). Furthermore, we applied specific pharmacological blockers for K_2P_3.1 (A293) and K_V_1.3 (ShK) channels and compared the current reduction in stimulated WT *versus* *K_2P_5.1^−^*^/*−*^ CD4^+^ T lymphocytes. Channel blockers had a significantly higher impact on *K_2P_5.1^−^*^/*−*^ cells, indicating a functional upregulation of K_2P_3.1 and K_V_1.3 on *K_2P_5.1^−^*^/*−*^ T lymphocytes (Figure 3C).

### 2.4. K_2P_5.1^−/−^ T Lymphocytes Display No Alterations in TCR-Dependent Signaling Pathways

Activation via the T cell receptor initiates the activation of multiple signaling pathways involving tyrosine phosphorylation cascades and Ca^2+^-dependent pathways [16]. We chose two key signaling enzymes involved in early (Zap70) and late events (ERK1/2) after TCR activation and assessed protein phosphorylation by specific antibodies. No significant differences were observed after TCR activation for Zap70 and ERK1/2 and after direct activation of downstream pathways by PMA for ERK1/2 (Figure 3D). Furthermore, Ca^2+^ imaging measurements using the Ca^2+^-sensitive dye Fura-2 revealed no significant alterations (Figure 3E). These experiments underline that K_2P_3.1 and K_V_1.3 are able to fully compensate K_2P_5.1 on a functional level.

### 2.5. Common Marmoset T Lymphocytes Functionally Express K_2P_5.1

**Figure 4:**
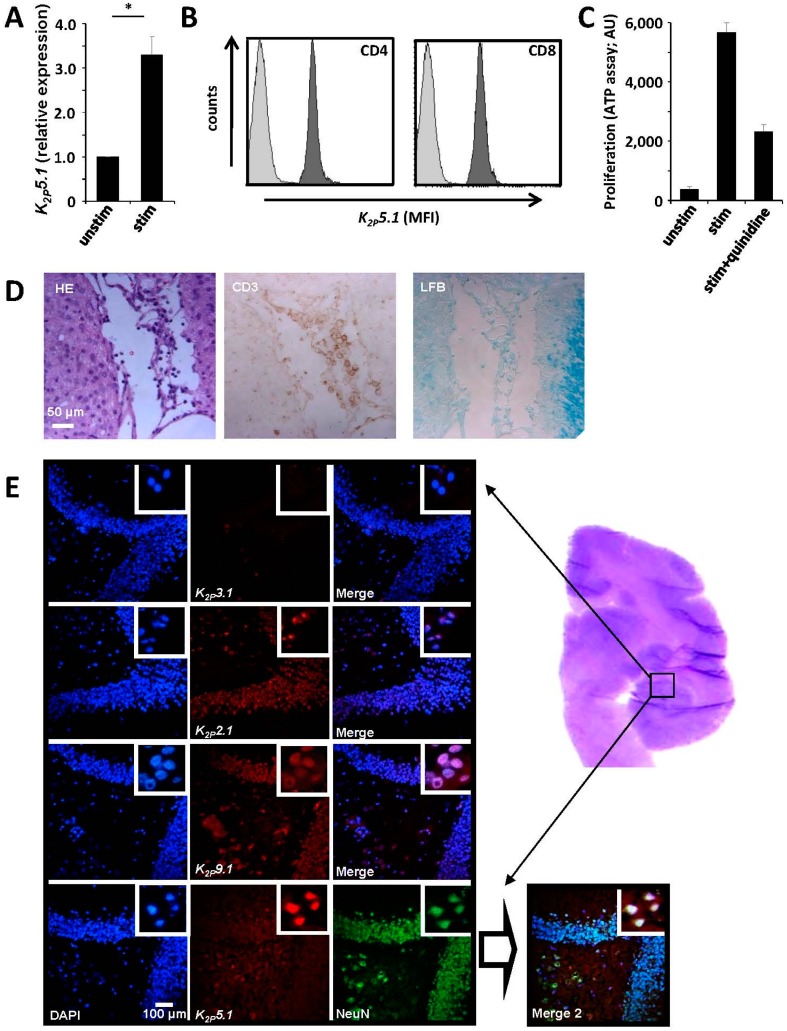
Expression of K_2P_5.1 channels in immune cells and CNS neurons in the common marmoset. (**A**) RT-PCR experiments show that CD4^+^ T lymphocytes from the common marmoset express *K_2P_5.1* in RT-PCR experiments, which is upregulated upon stimulation with PHA (*n* = 3); (**B**) CD4^+^ and CD8^+^ T lymphocytes express K_2P_5.1 protein as assessed by flow cytometry (one representative example out of three is shown); (**C**) The K_2P_5.1 inhibitor quinidine (20 µM) reduces the proliferation of stimulated marmoset CD4^+^ T lymphocytes (*n* = 3); (**D**) EAE lesions in the common marmoset characterized by HE, CD3 and LFB staining; (**E**) Brain samples from naive common marmosets were assessed by immunohistochemical staining for K_2P_2.1, K_2P_5.1 and K_2P_9.1. NeuN was used as a neuronal marker. The region of interest is displayed by HE staining on the right side. * = *p* < 0.05.

Our current findings demonstrate that *K_2P_5.1^−^*^/*−*^ mice show no obvious phenotype in autoimmune neuroinflammation due to a functional compensation by the potassium channels K_2P_3.1 and K_V_1.3. An alternative model for multiple sclerosis, EAE in the common marmoset (Callithrix jacchus), has been proposed especially for testing the efficacy of new potential drugs [17,18]. However, only very few commercial toolkits specifically designed for marmosets are currently available, making immunological or ion channel research difficult. Due to species homologies, both human and murine assays might potentially work with marmosets, which we could indeed confirm for a number of them (Table 1). Therefore, we were able to isolate marmoset CD4^+^ T lymphocytes and to stimulate them with phytohemagglutinin (PHA), leading to a significant upregulation of K_2P_5.1 channels (Figure 4A). Expression of K_2P_5.1 on protein level could also be confirmed by flow cytometric measurements for CD4^+^ and CD8^+^ T cells (Figure 4B). Application of the K_2P_5.1 inhibitor quinidine led to a reduced proliferation rate of marmoset T cells, hinting towards a functional relevance of K_2P_5.1 for marmoset T lymphocytes (Figure 4C). As the next step, we aimed to detect K_2P_5.1-expressing lymphocytes in EAE lesions in the common marmoset, which were characterized by HE, CD3 and Luxol fast blue (LFB) staining (Figure 4D). However, available anti-K_2P_5.1 antibodies provided no positive signals in paraffin-embedded tissue; hence, we have not been able to address this question due to technical limitations so far. Using cryo-fixed tissue of naive marmosets, we found positive signals for several K_2P_ channels (K_2P_2.1, K_2P_5.1, K_2P_9.1, but not K_2P_3.1) underlining the fact that this channel group might also be of functional importance in the marmoset brain. In summary, these results provide the first hint that marmosets might be of use for K_2P_ channel-related neuroimmunological research.

**Table 1:** Evaluation of commercial kits for marmoset research.

Commercial Kit	Company	Functional	Not Functional
CD4 non-human primate MACS kit	Miltenyi Biotec	X	
CD8 non-human primate MACS kit	Miltenyi Biotec		X
Mouse anti-human CD4 antibody (RPA-T4)	BioLegend	X	
Mouse anti-human CD8 antibody (RPA-T8)	BioLegend	X	
Human CD3/CD28 microbeads	Life Technologies		X
Mouse CD3/CD28 microbeads	Life Technologies		X
Phytohemagglutinin	Sigma-Aldrich	X	
ATP Assay	PerkinElmer	X	
Rabbit anti-human/mouse K_2P_5.1	Sigma-Aldrich	X	
Cytokine flow cytomix	BenderMed Systems		X
Human ELISA IFNγ, IL-2	eBioscience, RD Systems		X
Mouse ELISA IFNγ, IL-2	eBioscience, RD Systems		X
Human RT-PCR primers	Applied Biosystems	X	
Quinidine	Sigma-Aldrich	X	

Different commercial kits were evaluated with marmoset cells and are marked either as functional or as non-functional (“X” are set when applicable). See the Experimental Section for further details.

## 3. Discussion

Previously, K_2P_5.1 channels were shown to play an important role for T cell activation, both under physiological circumstances and under autoinflammatory conditions. In contrast to human T lymphocytes, we here demonstrate that K_2P_5.1, despite its expression under basal conditions, is not upregulated upon stimulation in murine T cells. Obviously, K_2P_5.1 seems to play a more pronounced role in human than murine T lymphocytes. Species-dependent differences have been reported especially for voltage-dependent potassium channels before [3]. Furthermore, genetic deletion of *K_2P_5.1* does not result in immune cell alterations *in vitro* or in the EAE model, most likely due to a compensatory upregulation of K_2P_3.1 and K_V_1.3 channels. These results were unexpected in light of previous results obtained from *K_2P_3.1^−^*^/*−*^ and *K_2P_9.1^−^*^/*−*^ mice that showed no compensatory upregulations of other channels, resulting in differences in immune cell functions. K_2P_5.1 was firstly discovered nearly 20 years ago [19]. It was initially named TWIK-related acid-sensitive K^+^ channel 2, due to the similar sensitivity to extracellular pH values compared to K_2P_3.1 [20]. However, it is now clear that K_2P_5.1 does not belong to the TASK, but rather to the phylogenetically- and structurally-different TALK subfamily of K_2P_ channels [21,22]. Despite obvious functional analogies of K_2P_3.1, K_2P_9.1 and K_2P_5.1, these molecular biological details should be kept in mind in order to avoid jumping to conclusions based on observations from one K_2P_ channel to another.

Different research groups have previously obtained experimental data from *K_2P_5.1^−^*^/*−*^ mice, which are often regarded to be more reliable than pharmacological studies due to a limited specificity of available K_2P_5.1 blockers. Besides its expression in immune cells, K_2P_5.1 function has been implicated in a variety of different cells and tissues, such as specific neuronal populations in the CNS, kidney epithelial cells or chondrocytes [21,23]. Early reports using newly generated *K_2P_5.1^−^*^/*−*^ mice described a contribution of K_2P_5.1 to cell volume regulation in mouse renal proximal tubule cells [24,25]. An involvement of K_2P_5.1 channels in regulatory volume decrease has subsequently been demonstrated for additional cell types using pharmacological approaches [24,26], while direct proof comparing WT and *K_2P_5.1^−^*^/*−*^ mice was only provided for *K_2P_5.1^−^*^/*−*^ lymphocytes [13]. K_2P_5.1 is involved in proximal tubule bicarbonate reabsorption, and *K_2P_5.1^−^*^/*−*^ mice show a secondary metabolic acidosis and hypotension [27]. In the CNS, K_2P_5.1 channel expression is normally restricted to respiratory center nuclei in the brainstem, and *K_2P_5.1^−^*^/*−*^ mice show disturbed respiratory responses to hypoxia and hypercapnia [28,29,30]. However, under pathophysiological acute ischemic conditions in an experimental model of cerebral ischemia, K_2P_5.1 is strongly upregulated on neurons where it supposedly contributes to the induction of neuronal apoptosis. Accordingly, *K_2P_5.1^−^*^/*−*^ mice had significantly reduced infarct volumes [31]. Interestingly, the functional contribution of K_2P_5.1 expression on T lymphocytes was evaluated for post-ischemic inflammatory reactions that deteriorate the experimental outcome in the used mouse model of transient middle cerebral artery occlusion. Adoptive transfer experiments of wild-type and *K_2P_5.1^−^*^/*−*^ T lymphocytes into *Rag1^−^*^/*−*^ mice prior to stroke induction showed no impact of K_2P_5.1 expression on T lymphocytes on stroke outcomes. These previous findings are in agreement with our current study showing that genetic deletion of *K_2P_5.1* is compensated by other potassium channels.

Our current findings show that the value of the classical murine MOG_35–55_-EAE model in C57BL/6 mice might be limited for a further functional and pharmacological assessment of K_2P_5.1 channels. The common marmoset displays a higher genetic homology with humans than mice (85% *versus* 40%) and might therefore be better suited as a future animal model for immunological assessment of the K_2P_5.1 channel-related pathology. Therefore, we conducted pilot experiments in the common marmoset providing first hints that marmosets might be worth further investigations in this context.

## 4. Experimental Section

### 4.1. Experimental Autoimmune Encephalomyelitis Induction and Evaluation

All animal experiments were approved by the local authorities (Landesamt für Natur, Umwelt und Verbraucherschutz NRW, Kirchhundem, Germany) and conducted according to the German law of animal protection. EAE was induced by immunization of 10–12-week-old female C57BL/6 (Charles River, Sulzfeld, Germany) or *K_2P_5.1^−^*^/*−*^ mice [24] with 200 µg MOG_35–55_ peptide (Biotrend, Cologne, Germany). MOG peptide was added to complete Freund’s adjuvants to obtain a 1– mg/mL emulsion, which was injected subcutaneously at the flank of deeply anesthetized mice. Pertussis toxin was injected on the day of immunization and 2 days later at a dose of 400 ng (Alexis, San Diego, CA, USA). Scoring was done by a blinded observer using the following score system: 0, no abnormality; 1, limp tail tip; 2, limp tail; 3, moderate hind-limb weakness; 4, complete paralysis of one hind-limb; 5, mild paraparesis; 6, paraparesis; 7, paraplegia; 8, tetraparesis; 9, quadriplegia or pre-moribund state; and 10, death. Animals with a score higher than 7 were euthanized, and the last score observed was included in the analysis until the end of the experiment. The cumulative EAE score was calculated by summing up each individual daily scores divided by the number of days.

### 4.2. Murine Cell Isolation and Culture

Spleens were isolated from age- and sex-matched mice (aged 8–12 weeks) or from immunized EAE mice at disease maximum (d15). Tissue was homogenized and strained through a 40 µm nylon filter. The homogenates were rinsed with washing medium (DMEM containing 1% FCS, 1% glutamine, 1% antibiotics) and shortly resuspended in erythrocyte lysis buffer (150 mM NH_4_Cl, 10 mM KHCO_3_, 0.1 mM EDTA; pH 7.3). For some experiments, thymic cells were isolated from 4–6 week old mice in a comparable fashion. Immune cell subsets were isolated using appropriate magnetic bead-based separation kits (CD4^+^ or CD8^+^ T cell isolation kit II, Miltenyi Biotec, Bergisch Gladbach, Germany). Cells were cultured in DMEM containing 10 mM HEPES, 25 μg/mL gentamicin, 50 μM β-mercaptoethanol, 5% FCS, 2 mM glutamine and 1% non-essential amino acids (Cambrex, Verviers, Belgium).

### 4.3. Immunological Analysis

Splenocytes were isolated either from naïve mice or from EAE mice at disease maximum. Cells were either stimulated with anti-CD3 (2 µg/mL) and anti-CD28 (1 µg/mL) antibodies or restimulated with 10 µg/mL MOG_35–55_ peptide. IFNγ, IL-17A, IL-2, IL-4 and IL-10 levels were assessed by enzyme-linked immunosorbent assay (ELISA, Ready-SET-Go! ELISA kit; eBioscience, Frankfurt, Germany).

For evaluation of cell proliferation, the amount of ATP in the supernatant after cell lysis was assessed as an indicator of cell proliferation using an ATPlite luminescence ATP detection assay system (PerkinElmer, Waltham, MA, USA) according to the manufacturer’s instructions and as described previously [32]. Luminescence was measured on an Infinite 200 PRO multimode microplate reader (Tecan, Männedorf, Switzerland), and the splenocyte stimulation index was calculated as cell abundance with stimulation divided by cell abundance without stimulation. Alternatively, 1 µCi of [3H]thymidine (Amersham, Piscataway, NJ, USA) was added for the final 24 h. Radioactivity was measured on a β-scintillation counter (TopCount NXT; PerkinElmer, Rodgau-Jügesheim, Hessen, Germany).

For flow cytometric evaluation of CNS-invading cells, mice were perfused transcardially with PBS to diminish contamination by leukocytes located within blood vessels. CNS tissue was dissociated mechanically, and mononuclear cells from the interface of a 30%–50% Percoll (GE Healthcare, Little Chalfont, Buckinghamshire, UK) density centrifugation gradient were further analyzed by flow cytometry. Cells were evaluated on a Gallios Flow Cytometer (Beckman Coulter, Krefeld, Germany).

For some experiments, peripheral blood mononuclear cells were obtained from the EUPRIM-NET program and used as described in Table 1.

### 4.4. Immunohistochemical Analysis

For histological evaluation of EAE, mice were transcardially perfused with PBS. Spinal cords were carefully excised and embedded in Tissue-Tek OCT optimal cutting temperature compound (Sakura Finetek, Torrance, CA, USA). To ensure that the same lumbar region was analyzed for all mice, transverse cryosections (10 mm thick) were cut and stained with HE or LFB according to standard protocols. Ten tissue sections separated by at least 40 mm were analyzed from five animals per group. Histological quantifications were assessed by an investigator blinded to treatment groups using an Axiophot 2 microscope (Carl Zeiss Microscopy, Jena, Germany) equipped with a charge-coupled device camera; images were analyzed with MetaVue research imaging software (Molecular Devices, Sunnyvale, CA, USA).

For fluorescence staining of marmoset brain slices, cryosections were postfixed in 4% paraformaldehyde for 10 min and incubated in blocking solution (PBS containing 5% bovine serum albumin, 1% donkey serum and 0.2% Triton X-100 surfactant). Slices were then incubated with antibodies against rabbit anti-mouse K_2P_3.1, rabbit anti-mouse K_2P_5.1, rabbit anti-mouse K_2P_9.1, rabbit anti-mouse K_2P_2.1 or rat anti-mouse NeuN (Millipore, Schwalbach, Germany) overnight at 4 °C. The secondary antibodies were Cy3 donkey anti-rabbit and Cy2 goat anti-rat (Dianova, Hamburg, Germany). Tissue was mounted with Prolong Gold antifade reagent containing DAPI (Life Technologies, Carlsbad, CA, USA). Negative controls were obtained by omitting either the primary or secondary antibody and revealed no detectable signal on subsequent analysis (data not shown).

### 4.5. Isolation of Human CD4^+^ T Lymphocytes

PBMC were isolated from peripheral blood of healthy individuals by density centrifugation using lymphocyte separation medium (Axis-Shield) according to the manufacturer’s instructions. CD4^+^ T lymphocytes were isolated using the CD4^+^ T cell isolation kit II (Miltenyi Biotec, Bergisch Gladbach, Germany) and stimulated by anti-human CD3 (OKT3, 2 µg/mL) and anti-human CD28 (28.2, 1 µg/mL, eBioscience, Frankfurt, Germany).

### 4.6. Flow Cytometry

Flow cytometric analysis was performed using the following anti-mouse antibodies (all from BioLegend, San Diego, CA, USA): anti-CD3 FITC (clone 17A2), anti-CD4 APC (RM4-5), anti-CD8a AF700 (53-6.7), anti-CD11b PE (M1/70), anti-CD19 PE/Cy7 (6D5), CD44 PE/Cy7 (IM7), CD62L PE (MEL-14), CD49d (R1-2), CD25 PE/Cy7 (PC61) and CD69 PE (H1.2F3). Cells were analyzed with a Gallios flow cytometer (Beckman Coulter, Krefeld, Germany).

### 4.7. Real-Time PCR

RNA was purified using TRIzol reagent (Life Technologies, Carlsbad, CA, USA), and RT-PCR was performed following standard protocols using TaqMan Gene Expression Assays (Life Technologies, Carlsbad, CA, USA) with specific primers for human K_2P_5.1 (Hs00186652_m1), mouse K_2P_3.1 (Mm04213388_s1), K_2P_5.1 (Mm00498900_m1), K_2P_9.1 (Mm02014295_s1), K_V_1.3 (Mm00434599_s1), K_Ca_3.1 (Mm00464586_m1) and eukaryotic 18S RNA (Hs9999901_s1). Data were calculated using Δ*C*_t_, ΔΔ*C*_t_ and relative quantification (2^−ΔΔ*C*t^).

### 4.8. Electrophysiology

Whole-cell electrophysiology was performed using isolated CD4^+^ T lymphocytes. All measurements were conducted in the whole-cell configuration of the patch-clamp technique 24 h after cell stimulation. Recording pipettes were filled with a solution containing (in mM): KCl, 140; HEPES, 5; MgCl_2_, 2; EGTA, 1; Na_2_-ATP, 1; GTP, 0.1; cAMP, 0.1. pH was set to 7.2 with KOH. Cells were continuously superfused with a bath solution containing (in mM): NaCl, 135; KCl, 5.4; HEPES, 5; MgCl_2_, 1; CaCl_2_, 1.8; and glucose, 10. pH was set to 7.4 with NaOH. Recordings were performed with an EPC-10 amplifier (HEKA Elektronik, Lambrecht, Germany). The resistance of the glass pipettes was 3–4 MΩ. The access resistance was in the range of 7–14 MΩ, and a series resistance compensation of more than 40% was used routinely. Measurements were either performed in the current or voltage clamp mode. The voltage protocol consisted of a depolarizing rectangular 500 ms pulse to +40 mV. A liquid junction potential of about 4 mV was measured and taken into account. Recordings were digitally analyzed using the Fitmaster software (HEKA Elektronik, Lambrecht, Germany).

### 4.9. Western Blots

For signaling analysis, cells were stimulated with anti-mouse CD3 (clone 145-2C11, 10 µg/mL) and anti-mouse CD28 (clone 37.51, 1 µg/mL, both eBioscience, Frankfurt,Germany) or 10 ng/mL PMA (Sigma-Aldrich, St. Louis, MO, USA) for 4 min. Cells were lysed for 30 min on ice in 30 µL lysis buffer (1% NP-40, 10% *N*-dodecyl-β-d-maltoside, 1 mM sodium monovanadate, 1 mM phenylmethanesulfonyl fluoride (PMSF), 50 µM TRIS, 10 mM NaF, 10 mM EDTA and 165 mM NaCl). Following 10 min of centrifugation (18,407× *g*, 4 °C), the supernatant was mixed with 7.5 µL sample buffer (20 mM TRIS, 10% glycerol, 0.05% bromophenol blue and 1% SDS) and heated for 5 min at 99 °C. Proteins were separated using a 10% SDS-PAGE and transferred to a nitrocellulose membrane. Membranes were blocked with 5% dry milk and probed with rabbit anti-murine K_2P_5.1 (Sigma-Aldrich), rabbit anti-p-ERK1/2 (Cell Signaling) or rabbit anti-p-Zap70 (Cell Signaling) diluted in 5% BSA and incubated overnight at 4 °C. Secondary antibody against rabbit was horseradish-peroxidase conjugated (Santa Cruz Antibodies, Dallas, TX, USA). The antibody reaction was detected by enhanced chemiluminescence reaction (ECL, Amersham Biosciences, Amersham, UK), and quantification was done using ImageJ software (V1.46r, originally developed by Wayne Rasband, now available as public domain). Anti-β-actin (Sigma-Aldrich, St. Louis, MO, USA) was used for loading controls.

### 4.10. Calcium Imaging

For calcium imaging experiments, T lymphocytes were isolated as described above. Analysis was performed in HEPES buffer containing 120 mM NaCl, 2.5 mM KCl, 1.25 mM NaH_2_PO_4_, 30 mM HEPES, 2 mM MgSO_4_, 10 mM glucose, pH 7.25, and osmolality was set to 305 mOsm/kg. Cells were loaded with 5 μM Fura-2 AM (Invitrogen, Karlsruhe, Germany) for 30 min at 37 °C. Fluorescence was measured with a TECAN infinite M200Pro fluorimeter (Tecan Group Ltd., Männedorf, Switzerland). Excitation was alternated between 340 and 380 nm, and emission was measured at 509 nm every 3 s. Either hamster anti-mouse CD3 and goat anti-hamster IgG antibodies (eBioscience, Frankfurt, Germany) or thapsigargin (0.1 µM; Sigma-Aldrich, St. Louis, MO, USA) were used for stimulation.

### 4.11. Statistical Analysis

All results are presented as the mean ± SEM. Statistical analysis was performed using Student’s *t*-test in the case of normally-distributed data or a Mann–Whitney test for parametric data without normality and equality of variance, as well as for non-parametric datasets. A one-way ANOVA with a Bonferroni *post hoc* test was used in the case of multiple comparisons for parametric data, and a Kruskal–Wallis ANOVA was used for non-parametric data. *p*-values < 0.05 were considered statistically significant.

## 5. Conclusions

Human T lymphocytes express K_2P_5.1 channels regulating T cell functions under physiological and pathological conditions. In contrast, *K_2P_5.1^−^*^/*−*^ mice display no significant alterations concerning T cell cytokine production, proliferation rates, surface marker molecules or signaling pathways resulting in an unaltered disease phenotype in an animal model of autoimmune inflammation. A compensatory upregulation of the potassium channels K_2P_3.1 and K_V_1.3 is responsible to counterbalance the deletion of *K_2P_5.1*. Preliminary experiments hint towards a potential use of the marmoset model for future research efforts as an alternative animal model.
